# Conventional chondrosarcoma with focal clear cell change: a clinicopathological and molecular analysis

**DOI:** 10.1111/his.13952

**Published:** 2019-09-13

**Authors:** Suk Wai Lam, Kirsten van Langevelde, Albert J H Suurmeijer, Arjen H G Cleven, Judith V M G Bovée

**Affiliations:** ^1^ Department of Pathology Leiden University Medical Centre Leiden The Netherlands; ^2^ Department of Radiology Leiden University Medical Centre Leiden The Netherlands; ^3^ Department of Pathology and Medical Biology University Medical Centre Groningen University of Groningen Groningen The Netherlands

**Keywords:** clear cell chondrosarcoma, conventional chondrosarcoma, *IDH1*, *IDH2*

## Abstract

**Aims:**

Clear cell chondrosarcomas are known to occasionally contain areas of low‐grade conventional chondrosarcoma; however, the opposite phenomenon has not yet been described. We identified five cases of conventional chondrosarcoma alongside clear cell chondrosarcoma. Here, we report on their clinicopathological and molecular characteristics, and investigate whether these hybrid lesions should be considered to be a collision tumour, conventional chondrosarcoma with clear cell change, or clear cell chondrosarcoma with extensive areas of conventional chondrosarcoma, as this has clinical implications.

**Methods and results:**

Clinicohistopathological features were characterised, immunohistochemistry was performed for H3 histone family member 3B (H3F3B), histone H3 trimethylated on lysine 27 (H3K27me3), and p53, and genetic alterations of *IDH1* (encoding isocitrate dehydrogenase 1), *IDH2* (encoding isocitrate dehydrogenase 2), *TP53* and *H3F3B* were evaluated. All five chondrosarcomas consisted predominantly of areas with conventional chondrosarcoma. Different grades were found [grade I (*n* = 1), grade II (*n* = 2), and grade III (*n* = 2)]. Up to 20% of the tumour consisted of classic features of clear cell chondrosarcoma. Gradual merging between both components was observed. Molecular analysis of conventional chondrosarcoma components revealed an *IDH1* c.395G>T, p.(Arg132Leu) mutation in two cases, and an *IDH1* c.394C>T, p.(Arg132Cys) mutation in one case, with identical *IDH* mutations in the clear cell chondrosarcoma counterpart (100%). Two cases were *IDH* wild‐type. In all cases, none of the components harboured *H3F3B* mutations. High‐grade tumours had an aggressive course, as three patients died of the disease.

**Conclusion:**

On the basis of clinicopathological characterisation and genetic alterations, it is suggested that these lesions should be considered to be conventional chondrosarcoma, with clear cell change. Pathologists should be aware of their existence to avoid confusion with clear cell chondrosarcoma, dedifferentiated chondrosarcoma, or chondroblastic osteosarcoma.

## Introduction

Chondrosarcomas constitute a group of malignant cartilaginous matrix‐producing tumours. This group encompasses neoplasms with diverse morphological features and clinical behaviours, with conventional chondrosarcoma being the most common subtype. Clear cell chondrosarcoma is a rare subtype of chondrosarcoma that is histologically characterised by cells with vesicular nuclei, surrounded by clear cytoplasm with distinct cytoplasmic membranes. Often, prominent trabeculae of woven bone are present. In these tumours, zones with conventional low‐grade chondrosarcoma may frequently (~50%) be encountered, either as large lobules of tumour cartilage surrounded by clear cells or as small inconspicuous foci among the clear cells.^1^, ^2^ The opposite phenomenon, i.e. areas of clear cell chondrosarcoma within conventional chondrosarcoma, has not been reported in the literature. Conventional central and clear cell chondrosarcoma are clearly two different entities: primary conventional central chondrosarcomas mainly develop in the thoracic, pelvic and long bones,^3^ whereas clear cell chondrosarcoma has a predilection for the epiphysis of long bones, particularly the proximal femur.^1^, ^2^ Furthermore, whereas curettage often leads to local recurrence with a risk of metastasis, which can occur after extended periods, *en‐bloc* excision is usually curative in patients with clear cell chondrosarcoma.^4^ The prognosis of conventional central chondrosarcoma depends greatly on histological grade, with 10‐year survival rates of 83% (grade I), 64% (grade II), and 29% (grade III), and metastases are often seen in high‐grade tumours (grade III: 70%).^5^ Besides distinct clinical and phenotypic features, different alterations are also found at the molecular level. *IDH1* (encoding isocitrate dehydrogenase 1) and *IDH2* (encoding isocitrate dehydrogenase 2) mutations are frequently (~50%) present in conventional central chondrosarcoma,^6^, ^7^ whereas clear cell chondrosarcomas lack *IDH1* and *IDH2* mutations (*n* = 17),^8^, ^9^ although a *H3F3B* (encoding H3 histone family member 3B) p.K36M mutation was found in one case.^10^ Here, we describe the clinicopathological features of five cases of chondrosarcoma with morphological features of conventional chondrosarcoma alongside areas of classic clear cell chondrosarcoma encountered in a bone tumour consultation practice. Molecular analysis of both components was performed in five cases to detect *IDH1*, *IDH2* or *H3F3B* mutations, in order to elucidate whether areas with clear cell chondrosarcoma morphology represent a phenotypic phenomenon occurring in conventional chondrosarcoma, represent a collision between two types of chondrosarcoma, or should be considered to be clear cell chondrosarcoma with extensive conventional chondrosarcoma areas.

## Materials and methods

### Case Selection

All conventional chondrosarcomas with clear cell chondrosarcoma features were encountered in a subspecialty consultation practice. Clinical and follow‐up data were retrieved from medical records. Surgical pathology specimens were routinely processed, and decalcified with EDTA or formic acid, after which slides were reviewed by an experienced bone and soft tissue pathologist (J.V.M.G.B.). Cases in which clear cell chondrosarcoma morphology constituted <50% of the specimen were included.

### Radiology

Magnetic resonance imaging (MRI) scans were reviewed in our tertiary tumour referral centre. MRI was performed externally in three cases (cases 1, 2, and 5) and in the Leiden University Medical Centre (LUMC) in two cases (cases 3 and 4). The LUMC MRI tumour protocol included T1‐weighted sequences, fluid‐sensitive sequences (T2 fat suppression), a T1 with fat suppression sequence, and a dynamic series after intravenous gadolinium contrast administration. For all cases, at least two imaging planes were available (axial and coronal, or axial and sagittal). Maximum tumour size was measured on sagittal or coronal sequences. Tumour signal intensity, perilesional bone marrow or soft tissue oedema and enhancement pattern were assessed. In addition, homogeneity was studied within the lesions to assess whether they contained components with different imaging characteristics on fluid‐sensitive sequences.

### Immunohistochemistry

Four‐micrometre deparaffinised formalin‐fixed paraffin‐embedded sections were incubated at 60°C. After antigen retrieval with Tris‐EDTA (pH 9.0) at 97°C for 30 min, representative slides containing both components were stained for H3F3B K36M (RM193, 1:2000; Sanbio, Uden, The Netherlands), histone H3 trimethylated on lysine 27 (H3K27me3) (C36B11, 1:10; Cell Signaling, Danvers, MA, USA) and p53 (DO‐7, 1:2000; Dako, Glostrup, Denmark) with the Omnis autostainer (Dako) and use of the Envision FLEX + detection kit (Dako).

### Mutation and Bioinformatic Analysis

Areas for microdissection and punching were carefully selected. To prevent contamination, areas composed of either conventional chondrosarcoma or clear cell chondrosarcoma features were selected. Areas where both components merged were avoided. DNA was isolated with a fully automated tissue preparation system (Siemens Healthcare Diagnostics, Tarrytown, NY, USA), as previously described.^11^ DNA isolation from frozen sections was performed with the Wizard Genomic DNA Purification Kit (Promega, Madison, WI, USA), according to the manufacturer's instructions. Mutation analysis of *IDH1* (exon 4), *IDH2* (exon 4) and *H3F3B* (exon 2) for cases 1–4 was performed with the multiplex Ampliseq‐based next‐generation sequencing (NGS) protocol, by use of the Custom AmpliSeq Cancer Hotspot Panels v3 (cases 2 and 3), v4 (case 1), and v4b (case 4).^12^ Bioinformatic analysis was performed as previously described.^12^ For case 5, Sanger sequencing was used for mutation analysis of *IDH1* and *IDH2*, as previously described.^13^ If an *IDH1* or an *IDH2* mutation in the conventional chondrosarcoma component was present, further analysis of the clear cell counterpart was performed.

## Results

### Clinical Characteristics

The clinical findings are shown in Table 1. Two patients were women and three patients were men. The median age at diagnosis was 63 years (range, 15–79 years). Tumour size ranged from 35 to 145 mm (mean, 89 mm). The presenting symptom was pain in most cases. Follow‐up data were available for all patients, with a mean of 20 months (range, 10–46 months). Three patients died of disease (time from diagnosis to death range, 10–46 months).

**Table 1:** Overview of clinical and pathological characteristics and results of molecular and immunohistochemical analyses

Case no.	Age (years)/sex	Primary localisation	Size (measured on MRI scan) (mm)	Grade on biopsy	Grade on resection/curettage	Amount of clear cell chondrosarcoma component (%)	Molecular analysis Conventional chondrosarcoma	Molecular analysis Clear cell chondrosarcoma	Follow‐up (months)	Outcome
1	79/M	Proximal tibia	90	Mainly necrosis At least I	III	15–20	T%: 50 *IDH1* c.395G>T, p.(Arg132Leu) Coverage: >2000 Allele frequency: 0.29 No *H3F3B* mutation	T%: 50 *IDH1* c.395G>T, p.(Arg132Leu) Coverage: >2000 Allele frequency: 0.03 No *H3F3B* mutation	10	DOD
2	15/F	Medial condyle of the femur	35	Biopsy not performed	I	5	No *IDH* mutation	Not performed	21	NED
3	46/M	Iliac bone	145	At least II	III	5–10	T%: 60 *IDH1* c.394C>T, p. (Arg132Cys) Coverage: >2000 Allele frequency: 0.36 *TP53* c.536A>G, p. (His179Arg) Coverage: >2000 Allele frequency: 0.73 No *H3F3B* mutation	T%: 40 *IDH1* c.394C>T, p. (Arg132Cys) Coverage: 582 Allele frequency: 0.08 *TP53* c.536A>G, p. (His179Arg) Coverage: 259 Allele frequency: 0.59 No *H3F3B* mutation	15	DOD
4	67/F	Nasal septum	35	I–II	II	5	No *IDH* mutation	Not performed	7	NED
5	63/M	Proximal femur	140	II and suspicious for dedifferentiation	II	5	T%: 60 *IDH1* c.395G>T, p.(Arg132Leu)	T%: 60 *IDH1* c.395G>T, p.(Arg132Leu)	46	DOD

DOD, Died of disease; F, Female; H3F3B, H3 histone family member 3B; IDH, Isocitrate dehydrogenase; M, Male; MRI, Magnetic resonance imaging; NED, No evidence of disease; T%, Tumour percentage.

### Radiological Characteristics

**Figure 1:**
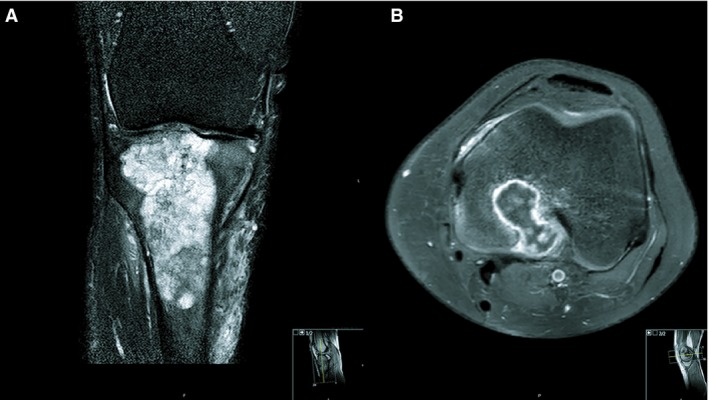
**A**, Magnetic resonance imaging of case 1. A coronal T2 SPIR sequence of the right knee shows a multiloculated lesion in the proximal tibia epimetaphysis. The lesion contains T2 hyperintense cartilage nodules, which are typical for chondroid tumours. The extension into the epiphysis (in this case up to the subchondral bone plate) is not typical for a chondroid tumour, which is usually contained in the metaphysis. Small internal foci of low signal intensity are noted, and are in keeping with chondroid matrix calcifications. Perilesional bone marrow oedema and soft tissue oedema are present. **B**, Magnetic resonance imaging of case 2. Axial T1 SPAIR after administration of gadolinium contrast in the left distal femur shows a typical septonodular or ‘rings‐and‐arcs’ enhancement in this cartilaginous tumour. The lesion extends into the epiphysis and through the posterior cortex into the soft tissues. Perilesional bone marrow oedema is present.

Three cases occurred in the long bones (case 1, proximal tibia; case 2, distal femur; case 5, proximal femur). The tumours had a metaphyseal location with extension into the epiphysis and diaphysis (case 1; Figure 1A), a metadiaphyseal location (case 5), and an epiphyseal location with extension into the metaphysis (case 2; Figure 1B). The iliac bone was involved in case 3. In case 4, the tumour was located in the orbit and maxillary sinus, presented with a soft tissue component, and concerned a recurrence of a chondrosarcoma of the nose septum. All tumours were homogeneous and mainly hyperintense on T2‐weighted/fluid‐sensitive MRI sequences. After intravenous gadolinium contrast administration, a typical pattern of septonodular enhancement was noted. None of the cases showed components of different signal characteristics within the lesions such as to suggest an area of clear cell chondrosarcoma. In four cases (cases 2–5), soft tissue involvement was present. Perilesional bone marrow oedema was present in four cases (cases 1–3 and 5). High‐grade chondrosarcoma was the only radiological differential diagnosis in four cases (cases 1 and 3–5). However, in case 2, locally aggressive chondroblastoma and clear cell chondrosarcoma were also considered, given the epiphyseal location, which is unusual for conventional chondrosarcoma.

### Histopathological Characteristics

Four patients (cases 1, 3, 4, and 5) underwent a surgical resection with a biopsy prior to the resection, and one patient (case 2) underwent a curettage. All biopsies revealed a conventional chondrosarcoma component, and were graded according to Evans on a scale of I–III, based on nuclear size, hyperchromasia, mitotic figures, and cellularity (Table 1).^14^, ^15^ No clear cell chondrosarcoma component was observed. In case 5, dedifferentiated chondrosarcoma was considered, given the presence of areas with abrupt transition to atypical spindle cells. Review of the primary chondrosarcoma of the nose septum (case 4) did not reveal areas with clear cell chondrosarcoma morphology.

**Figure 2:**
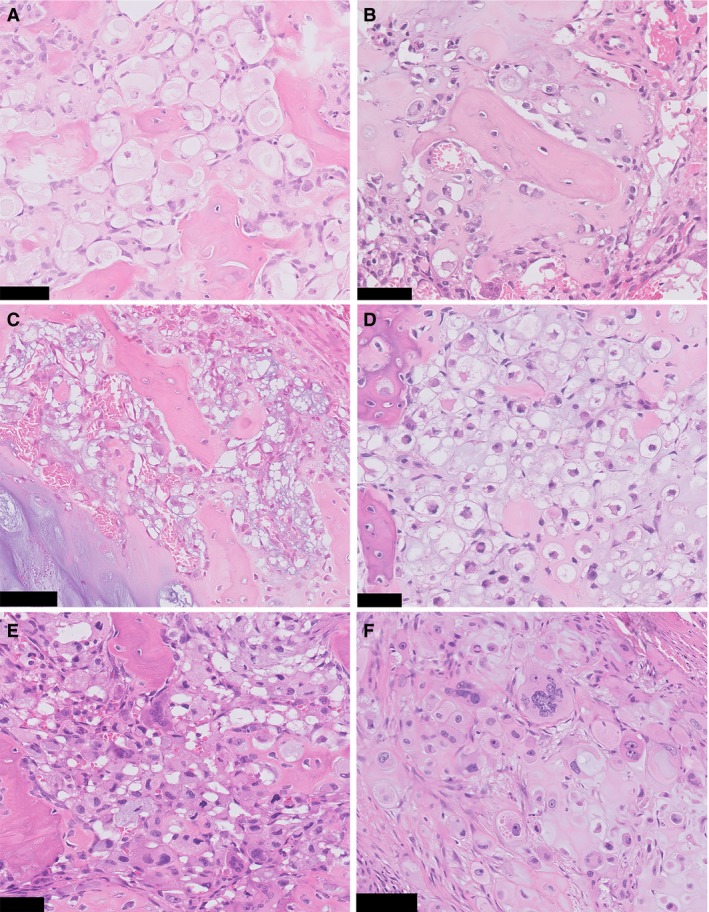
Clear cell chondrosarcoma areas with variable degrees of nuclear atypia. **A**, Clear cells are admixed with trabeculae of woven bone, resembling clear cell chondrosarcoma. Areas with large clear cells with distinct cytoplasmic membranes are encountered in most cases. Nuclei are small, regular, and without prominent atypia (case 1). **B**–**D**, Cells with slightly enlarged and hyperchromatic nuclei can be present (case 2, case 4, and case 5, respectively). Note the regular deposition of woven bone that is characteristic of clear cell chondrosarcoma. **E**, Severe atypia can be seen. Cells with enlarged, irregular and hyperchromatic nuclei are admixed with woven bone and osteoclast‐like giant cells (case 3). **F**, Occasionally, cells with bizarre and monstrous nuclei are distributed between more typical cells with round nuclei and prominent nucleoli, surrounded by abundant eosinophilic cytoplasm (case 3). Scale bar: 50 μm (**A**–**E**), 100 μm (**F**).

**Figure 3:**
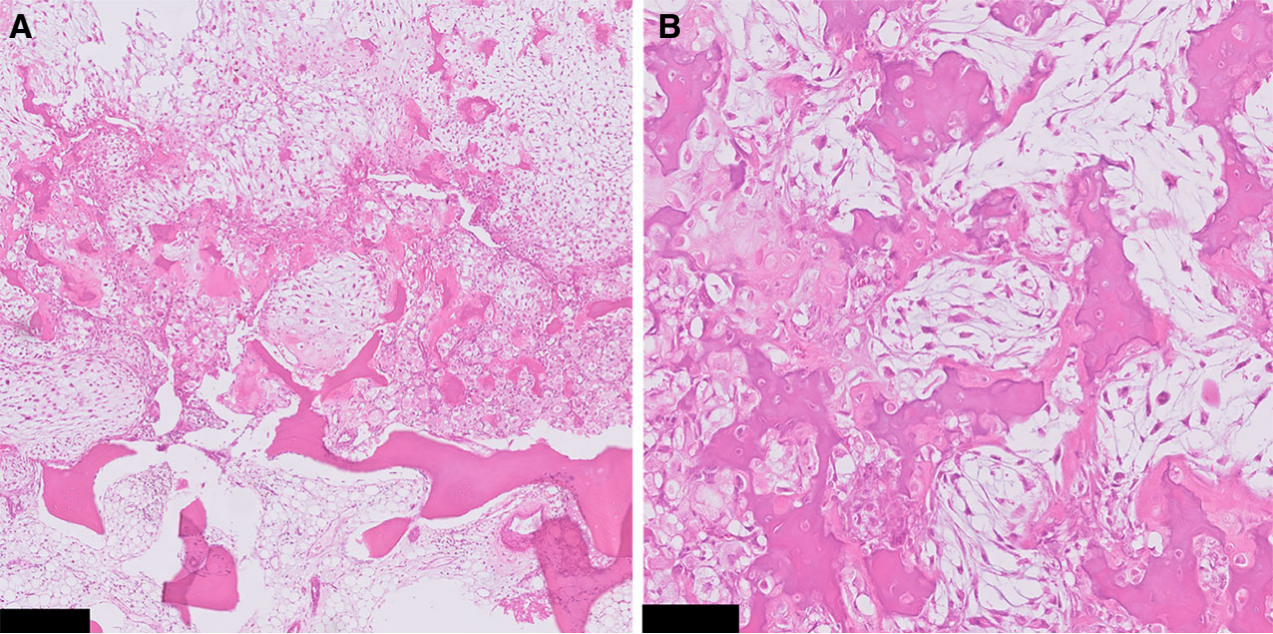
**A**, Haphazardly arranged areas with clear cell chondrosarcoma morphology and conventional chondrosarcoma. **B**, At high power, both components gradually merge together, as opposed to dedifferentiated chondrosarcoma (case 1). Scale bar: 250 μm (**A**), 100 μm (**B**).

All curettage and resection specimens showed morphological features of both conventional chondrosarcoma and clear cell chondrosarcoma. Conventional chondrosarcoma components were graded according to Evans. One tumour was grade I (case 2), two were grade II (cases 4 and 5), and two were grade III (cases 1 and 3). The clear cell components were composed of cells with abundant eosinophilic to clear cytoplasm with distinct membranes. Overall, nuclei were round and centrally located, with a prominent central nucleolus (Figure 2A). Areas of atypia were occasionally observed, and ranged from cells with slightly enlarged, hyperchromatic nuclei to cells with bizarre and monstrous nuclei (Figure 2B–F). The degree of atypia varied between cases, and was most prominent in case 3. Furthermore, tumour cells were admixed with a regular deposition of trabeculae of woven bone, and numerous osteoclast‐like giant cells. The amount of clear cell chondrosarcoma areas varied between cases, and ranged between 5% and 20%. Noticeably, these areas were scattered throughout the specimens and did not show abrupt transition, but rather merged with the conventional chondrosarcoma components (Figure 3).

### Molecular Analysis

**Figure 4:**
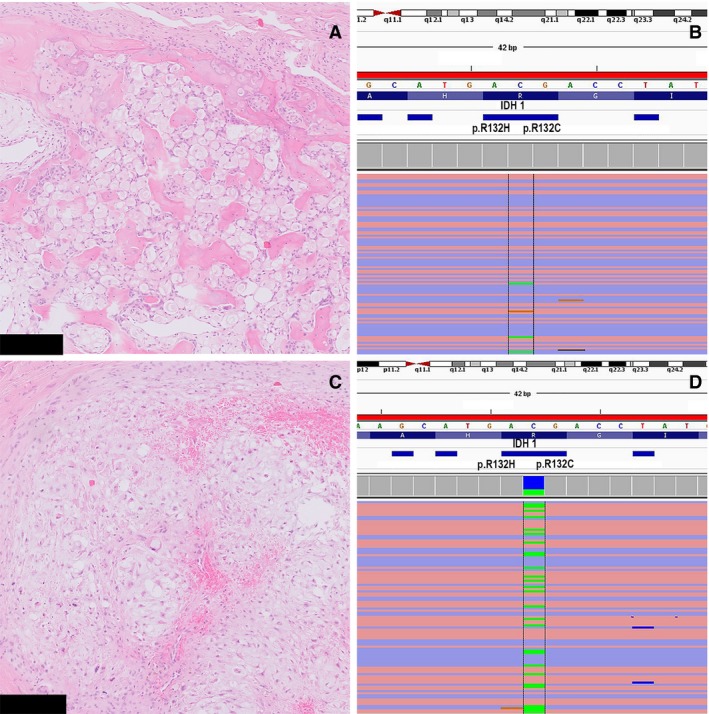
**A**, Microdissected clear cell chondrosarcoma area used for next‐generation sequencing (NGS). **B**, NGS showing the presence of the *IDH1* c.395G>T, p.Arg132Leu mutation (allele frequency: 0.03). **C**, Microdissected conventional chondrosarcoma area used for NGS. **D**, NGS showing the presence of an identical *IDH1* c.395G>T, p.Arg132Leu mutation (allele frequency: 0.29) (case 1). Scale bar: 250 μm (**A**,**C**).

Molecular analysis of the conventional chondrosarcoma component for *IDH1* and *IDH2* was possible in all cases. *IDH1* mutations were found in three cases, i.e. *IDH1* c.395G>T, p.(Arg132Leu) in case 1 (Figure 4) and case 5, and *IDH1* c.394C>T, p.(Arg132Cys) in case 3. Cases 2 and 4 showed no *IDH* mutation. The clear cell counterpart of *IDH* mutant conventional chondrosarcoma could be evaluated in all three cases. Case 3 showed suboptimal sequence metrics, and analysis was therefore repeated. All samples showed identical *IDH* mutations. Molecular analysis for *H3F3B* was performed for cases 1 and 3, and showed no mutation. Additional mutations in *TP53* c.536A>G, p.(His179Arg) were found in both components of case 3 (Table 1).

### Immunohistochemistry

**Figure 5:**
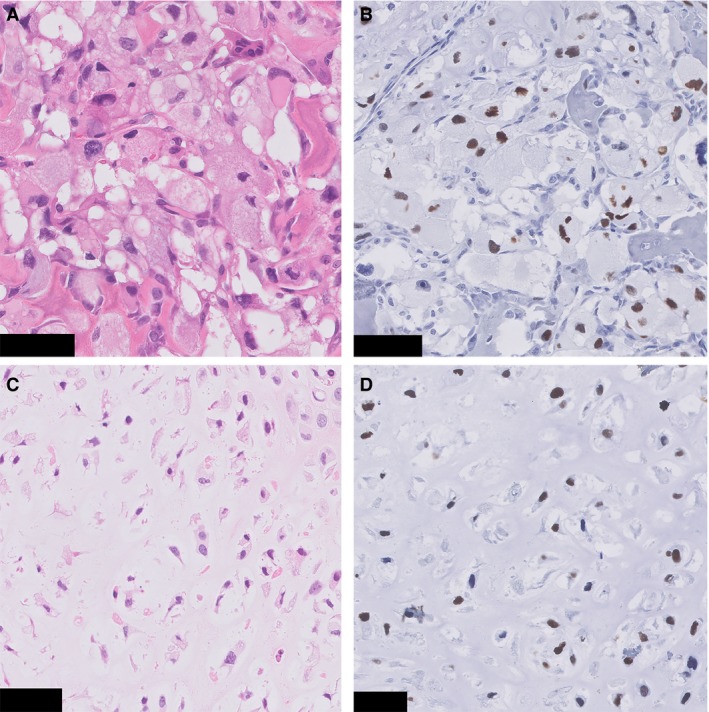
p53 immunohistochemistry (case 3). **A**, Clear cell chondrosarcoma component. **B**, Immunohistochemistry for p53 in the same tumour region shows overexpression in the tumour cells. **C**, Conventional chondrosarcoma area. **D**, Immunohistochemistry for p53 shows overexpression in the tumour cells. Scale Bar: 50 μm (**A**–**D**).

Immunohistochemistry for H3K27me3, H3F3B K36M and p53 was performed for both components. Because a recent publication showed deficiency of H3K27me3 in a subset of dedifferentiated chondrosarcomas,^16^ immunohistochemical analysis of H3K27me3 was performed to rule out this possibility. No case showed loss of expression. No tumour showed positive staining for H3F3B K36M, whereas the external control was positive. In case 3, immunohistochemistry for p53 showed, in both components, a mutant staining pattern (Figure 5), whereas in the remaining cases a wild‐type staining pattern was observed.

## Discussion

In this article, we present the clinical and histopathological characteristics of five cases in which we observed clear cell chondrosarcoma areas adjacent to conventional chondrosarcoma. Molecular and immunohistochemical analyses were performed to investigate whether these hybrid tumours should be considered to be part of a collision tumour, a conventional chondrosarcoma with clear cell change, or clear cell chondrosarcoma with extensive areas of conventional chondrosarcoma.

Clear cell chondrosarcoma was first described by Unni *et al*. in 1976,^1^ and it has been noticed that these tumours may contain areas of conventional low‐grade chondrosarcoma. This tumour has low malignant potential, and *en‐bloc* resection with clear margins is usually curative.^1^, ^2^, ^17^ Whereas *IDH1* and *IDH2* mutations can be encountered in ~50% of conventional chondrosarcomas,^6^ *IDH* mutations are absent in clear cell chondrosarcoma.^8^, ^9^ Recently, an *H3F3B* p.K36M mutation, which is typically found in chondroblastoma of bone,^10^ was found in one clear cell chondrosarcoma.^18^ Although conventional chondrosarcoma may show a variety of histological features, depending on histological grade, there are, as yet, no reports of conventional chondrosarcoma admixed with areas of clear cell chondrosarcoma features in the literature, to the best of our knowledge. Histopathological analysis of the five cases revealed a tumour configuration consisting of 5–20% of clear cell chondrosarcoma areas with variable degree of atypia, gradually merging with the conventional chondrosarcoma component. Furthermore, four of the five tumours were located at the predilection sites for conventional chondrosarcoma, and behaved as would be expected on the basis of histological grade of the conventional chondrosarcoma component. Also, identical *IDH* and *TP53* mutations were present in both components of case 3. In two cases, conventional chondrosarcoma areas lacked *IDH* mutations, which could be expected, given the frequency of *IDH* mutations in conventional chondrosarcoma,^6^ and none of the cases showed an *H3F3B* mutation. The MRI characteristics in four of the five cases were in keeping with conventional chondrosarcoma, and did not show a separate clear cell chondrosarcoma component within the tumours. In case 2, the epiphyseal location of the tumour was the only finding that pointed towards clear cell chondrosarcoma. Our results therefore suggest that clear cell chondrosarcoma areas in conventional chondrosarcoma represent a morphological phenomenon, rather than a collision between two tumour types or clear cell chondrosarcoma with extensive areas of conventional chondrosarcoma. An exact cut‐off of the volume of the clear chondrosarcoma‐like component within conventional chondrosarcoma can only be determined when more cases are presented after more awareness has been raised.

Importantly, the presence of woven bone within the clear cell areas combined with features of a cartilage tumour can mislead the pathologist. In dedifferentiated chondrosarcoma, areas with neoplastic bone formation can be seen. The architectural composition should be discriminating in these cases: in dedifferentiated chondrosarcoma an abrupt transition is seen between both components,^19^ whereas in the current cases clear cell chondrosarcoma areas gradually merge with conventional chondrosarcoma areas. Also, in dedifferentiated chondrosarcoma the dedifferentiated component clearly shows high‐grade features, whereas the present cases have a very specific clear cell chondrosarcoma morphology with variable nuclear atypia. Recently, loss of H3K27me3 was described in 32% of dedifferentiated chondrosarcomas, and was restricted to the dedifferentiated component.^16^ In the present series, all cases showed retention of H3K27me3 in the clear cell areas, which is in support of these tumours not belonging to this distinct subset of dedifferentiated chondrosarcomas with H3K27me3 deficiency.

Similarly, on the basis of the presence of bone formation, chondroblastic osteosarcoma can also enter the differential diagnosis. The presence of conventional chondrosarcoma areas with limited nuclear atypia should lead to the correct diagnosis, because, in chondroblastic osteosarcoma, the nuclear atypia in the cartilaginous areas is usually abundant. Moreover, the trabeculae in clear cell chondrosarcoma show osteoblastic rimming, and this is usually much more regular and ‘rhythmic’ than the more irregular deposition in chondroblastic osteosarcoma. The distinction can be challenging, but is crucial, as high‐grade osteosarcoma requires different treatment.^20^ Molecular analysis of *IDH* can be helpful, as the presence of this mutation strongly favours chondrosarcoma over chondroblastic osteosarcoma.^21^

It should be noted that the sequence metrics of case 3 were suboptimal, owing to a long period of decalcification with formic acid, but identical *IDH1* and *TP53* mutations were found in both components, also after repetition of NGS. It is of note that the allele frequency of *IDH* mutant reads was relatively low in clear cell areas, as compared with areas with conventional chondrosarcoma and as compared with the *TP53* variant. Therefore, we cannot completely rule out the possibility that the clear cell chondrosarcoma area lacks the *IDH* mutation. Unfortunately, for this specific mutation, no antibody is available for immunohistochemical confirmation.^22^ An explanation for the low allele frequency could be the higher contamination with normal cells. Even though we used microdissection, the clear cell chondrosarcoma areas were scattered throughout the specimen, and tumour cells were intermingled with giant cells and stromal cells, which may have decreased the allele frequency in the clear cell chondrosarcoma areas as compared with the more homogeneous conventional chondrosarcoma areas. Also, as heterogeneity can often be present in tumours,^23^ this can contribute to the lower allele frequency in the clear cell component. The fact that we confirmed the presence of p53 overexpression by using p53 immunohistochemistry in both components of case 3 further supports the clonal relationship between the clear cell chondrosarcoma and the conventional chondrosarcoma areas.

Although, in our experience, conventional chondrosarcoma areas constitute a minor component of clear cell chondrosarcoma, no exact percentages for the amount of conventional chondrosarcoma areas within clear cell chondrosarcoma are available.^1^, ^2^ Therefore, we cannot completely rule out the possibility that the two *IDH* wild‐type cases represent clear cell chondrosarcoma with an abundant conventional chondrosarcoma component. This is especially relevant for case 2, as it involved a young patient with an epiphyseal tumour with a low‐grade chondrosarcoma component. The radiology of case 4 was more in line with conventional chondrosarcoma, and high‐grade (grade II) conventional chondrosarcoma was morphologically encountered. As no specific molecular alterations have been reported in clear cell chondrosarcoma, at present this issue cannot be further resolved.

In conclusion, we report five cases of conventional chondrosarcoma with clear cell chondrosarcoma features, in which additional clinical, histopathological and molecular characterisation of both components suggest that this represents a morphological phenomenon. Clear cell chondrosarcoma features are rarely encountered in conventional chondrosarcoma, but should not prevent the pathologist from establishing the diagnosis of conventional chondrosarcoma. Most importantly, these lesions should not be confused with clear cell chondrosarcoma, dedifferentiated chondrosarcoma, or chondroblastic osteosarcoma, as treatment and outcome differ significantly.

## Conflict of interest

The authors state that they have no conflicts of interest.

## Author contributions

The study was designed, written and reviewed by S. W. Lam and J. V. M. G. Bovée. All authors contributed to data collection, data analysis, and interpretation. The manuscript was approved by all authors.

## Ethics approval

All samples were coded according to the Dutch code of proper secondary use of human material as accorded by the Dutch Society of Pathology (Federa), and as approved by the LUMC ethical board (B17.039).
